# Low-Grade Fibromyxoid Sarcoma of the Abdominal Wall: A Clinical Case Report

**DOI:** 10.7759/cureus.35699

**Published:** 2023-03-02

**Authors:** Milton Alberto Muñoz-Leija, Marion Carolina Alemán-Jiménez, Heliodoro Plata-Álvarez, Grecia Menes-Ramírez

**Affiliations:** 1 General Surgery, Hospital General de Zona 6, Instituto Mexicano del Seguro Social, San Nicolas de los Garza, MEX; 2 Human Anatomy, Universidad Autonoma de Nuevo Leon, School of Medicine, Monterrey, MEX

**Keywords:** soft tissue surgery, soft tissue tumour, low-grade fibromyxoid sarcoma, abdominal wall surgery, abdominal wall tumor

## Abstract

Low-grade fibromyxoid sarcoma (LGFMS) is a soft tissue neoplasm that occurs preferentially in young, male adults as a slowly growing, asymptomatic mass. According to current literature, the most common anatomical sites where it occurs are the trunk and lower extremities, especially the thigh, perineum, and groin. The risk factors are still unknown. Surgical intervention (simple resection and wide excision) is nowadays considered the best treatment option; however, patients require a long follow-up due to the high recurrence and metastasis rates. We present a low-grade fibromyxoid sarcoma case located in the abdominal wall of a female Hispanic patient.

## Introduction

Soft tissue tumors are uncommon tumors, accounting for only approximately 1% of cancers in adults [1]. Low-grade fibromyxoid sarcoma (LGFMS), also known as Evans tumor or hyalinizing spindle cell tumor with giant rosettes, is a distinctive type of soft-tissue sarcoma that is typified by a deceptively benign histologic appearance and very indolent but fully malignant behavior [2]. LGFMS occurs preferentially in young male adults [3] and presents as a slowly growing asymptomatic mass on the lower extremities, usually the thigh, followed by the groin, perineum, and trunk. Other sites include the neck, axilla, chest wall, shoulder, inguinal region, and rarely the mediastinum, retroperitoneum, mesentery, and pelvis. Most lesions are localized to the deep soft tissues, including the skeletal muscle [4,5]. In this paper, we report a rare case of low-grade fibromyxoid sarcoma of the anterior abdominal wall in a young female adult.

## Case presentation

**Figure 1:**
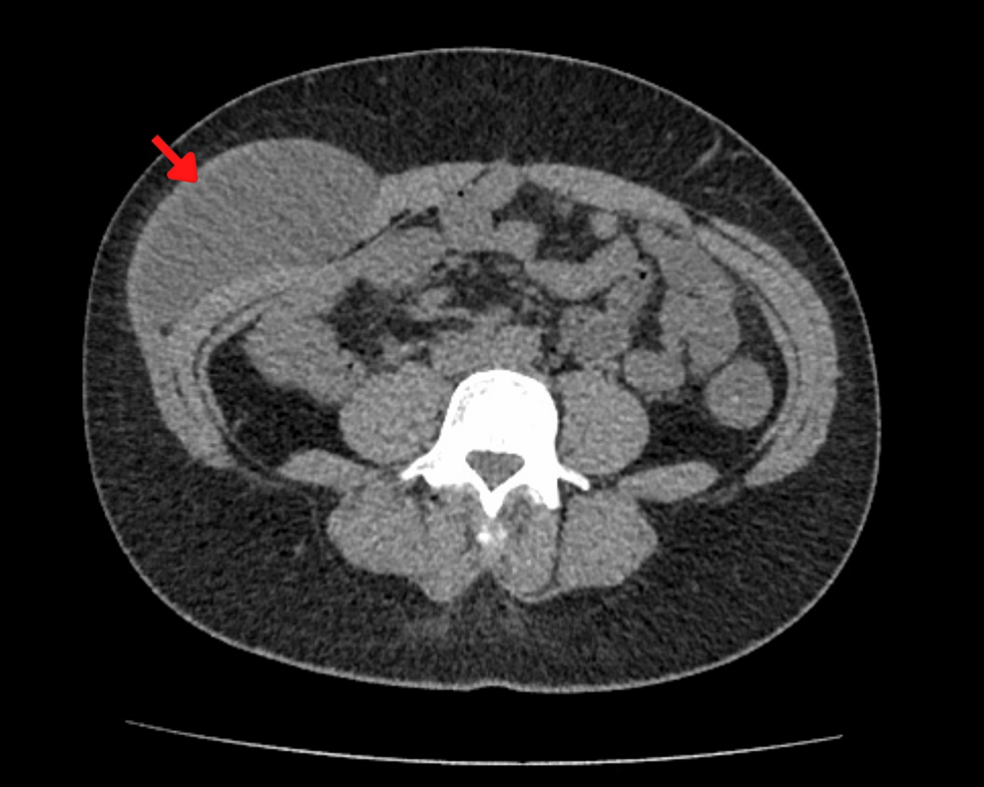
An axial view of abdominal computed tomography shows a mass between the external and internal oblique muscles on the right side (red arrow)

A 32-year-old female patient with no significant past medical history presented to the general surgery outpatient clinic with a three-year history of an enlarging mass in the right flank region. Upon physical examination, the mass was well-delimited, firm, attached to deep anatomical planes, and of soft consistency. Weight loss, fever, or pain were denied. Abdominal ultrasound revealed a 129 x 50 mm solid nodular mass with heterogeneous echogenicity within the soft tissue. An axial abdominal computed tomography was then performed, revealing a 10 x 6 x 18 cm cystic mass located between the external and internal oblique muscles on the right side (Figure 1).

**Figure 2:**
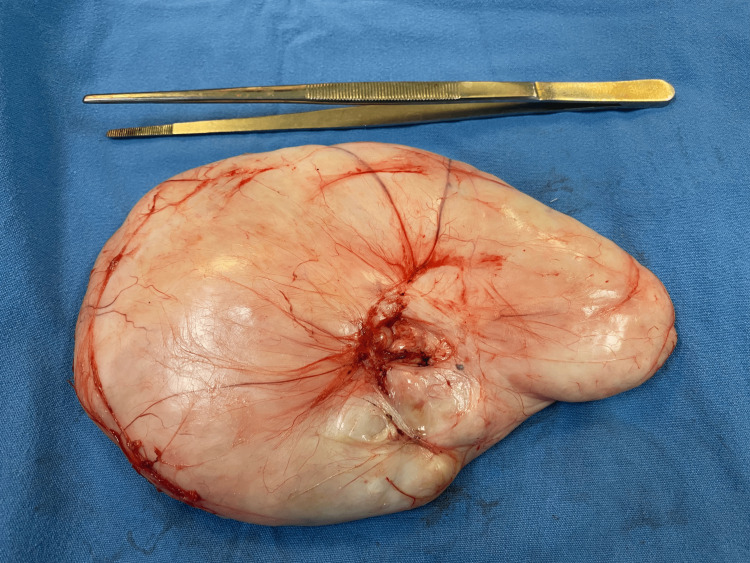
Fresh resection specimen of the mass

**Figure 3:**
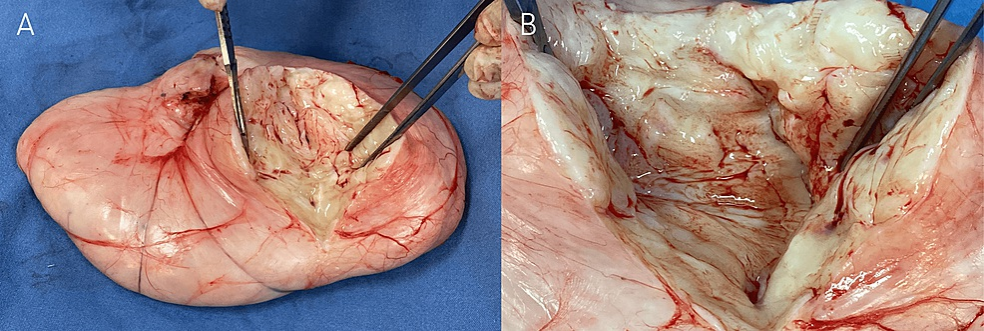
Internal content of the resected mass (A) A mass filled with a tan-white solid material; (B) A close-up of the internal content

The patient underwent a laboratory workup that included a white blood cell count (6.9 × 10⁹/L), hemoglobin level (13.6 g/dL), hematocrit (40.6%), platelet count (328 × 10⁹/L), and glucose level (88 mg/dL), all of which were within the reference range. Surgical treatment was decided. A paramedian incision followed by a simple incision over the external oblique muscle was performed, then the mass was removed through a wide surgical excision with a safety margin of 2 cm. Intraoperative findings confirmed a solid mass measuring 18 x 10 cm, located between the external and internal oblique muscles of the abdomen without attachment to them. No abdominal wall reconstruction was needed, and a simple running closure using Polyglactin 910 was performed. On gross examination, the resected specimen consisted of a smooth, oval mass with congested capillaries (Figure 2) filled with a tan-white solid material (Figures 3A, 3B).

**Figure 4:**
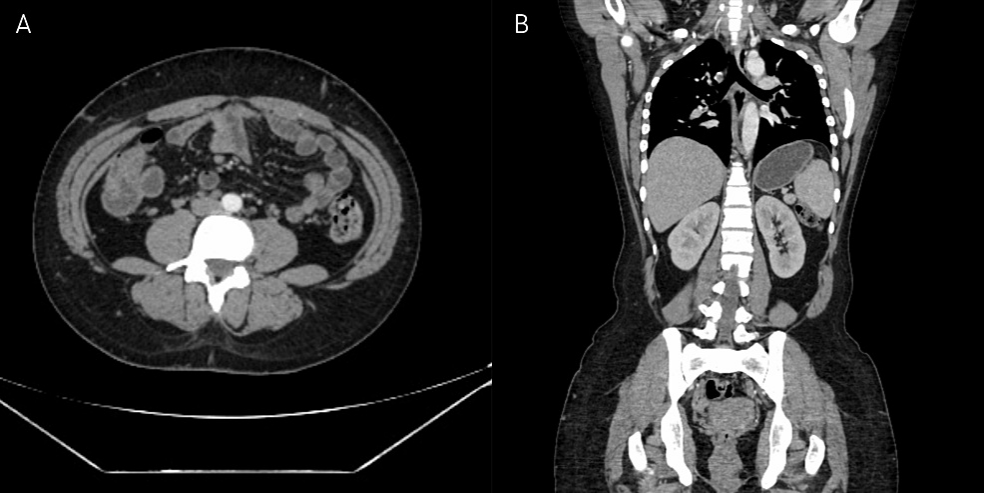
Abdominal CT during follow-up Multiple abdominal CT images: axial view (A), and coronal view (B), demonstrating no evidence of local recurrence or metastasis.

Histologic analysis revealed a well-delimited neoplasm with fusiform fibroblastic cells with swirling growth patterns and collagen rosettes in a myxoid stroma. Immunohistochemistry demonstrated positivity for mucin-4 (MUC-4) and epithelial membrane antigen (EMA), confirming the diagnosis of low-grade fibromyxoid sarcoma. Consequently, the patient was referred to oncology for follow-up. After two years of follow-up, imaging showed no evidence of local recurrence or metastasis (Figures 4A, 4B).

The patient currently remains under surveillance.

## Discussion

Low-grade fibromyxoid sarcoma was first described by Evans in 1987, who subsequently described additional cases in 1993 [4]. LGFMS, which commonly occurs in patients between six and 52 years of age (median of 29 years), is more prevalent in men and tends to range from 1.5 to 16 cm in size (maximum diameter) [2]. In our patient, the age at which LGFMS presented was in accordance with the literature, but the patient’s sex and tumor size were not within the usual range. The anatomic distribution of the LGFMS is predominant in the thigh and trunk. Other sites described in reports include the neck, chest wall, back, mesentery, hand, retroperitoneum, leg, and abdominal wall. In this case, the tumor was located on the abdominal wall, which, according to current literature, is not a common anatomic site. To date, 13 cases of LGFMS in the abdominal wall have been reported in a total of 10 articles, of which six were female and seven were male [2, 6-14] (Table 1).

**Table 1:** Low-grade fibromyxoid sarcoma of the abdominal wall (cases reported in the literature) The table shows the cases of low-grade fibromyxoid sarcoma of the abdominal wall reported up to date, specifying the sex, age, and diameter of the specimen.

Author	Year	Country	Sex	Age (years)	Largest diameter of the tumor (cm)	Surgical Procedure
Van den Bossche et al. [6]	2000	Belgium	Female	38	27	Simple resection
Oda et al. [7]	2004	Japan	Female	47	4	Simple resection
Guillou et al. [8]	2007	Switzerland	Male	35	Unknown	Simple resection
Guillou et al. [8]	2007	Switzerland	Female	44	2.5	Simple resection
Meng et al. [9]	2008	China	Male	41	4	Unknown
Evans et al. [2]	2011	United States	Female	22	Unknown	Simple resection
Evans et al. [2]	2011	United States	Male	21	3	Simple resection
Singh et al. [10]	2012	India	Female	30	10	Wide excision
Hashimoto et al. [11]	2016	Japan	Male	74	2	Wide excision
Sakaguchi et al. [12]	2016	Japan	Male	8	4	Simple resection
Ud Din et al. [13]	2018	United States	Female	55	Unknown	Simple resection
Ud Din et al. [13]	2018	United States	Male	5	8.5	Simple resection
Ronen et al. [14]	2022	United States	Male	45	9.2	Unknown

To the best of our knowledge, this is the first case of an Evans tumor located on the abdominal wall reported in our country.

Rates of local recurrence and distant metastasis have been reported in several articles, ranging from 9% to 73% for local recurrence in a period of up to 15 years and 6% to 45% for metastasis, which may develop in a period of up to 45 years [2,12]. The treatment reported in the literature that continues to be widely accepted among different studies and case reports is surgical intervention (including wide excision with a safety margin and simple resection without a safety margin). However, incompleteness of the excision margin is a risk factor for recurrence and metastasis [12]. In our patient, management with wide excision with a safety margin of 2 cm has not shown local recurrence or metastasis in a period of two years.

Although to date the recommended management for LGFMS is complete surgical removal with a margin of safety and long-term follow-up, more long-term studies must be carried out to assess the effectiveness of extensive surgery with its subsequent follow-up. The use of chemotherapy and radiotherapy remains controversial within the current literature [12].

## Conclusions

Low-grade fibromyxoid sarcoma of the abdominal wall is an extremely rare neoplasm in an unusual location, with only a few cases reported in the literature. Surgical intervention is currently the accepted treatment for LGFMS, with wide excision being the preferred procedure; however, long-term studies must be carried out to assess its effectiveness. Despite its benign appearance, clinicians must inform patients with LGFMS of the imperative need for strict subsequent follow-ups due to high local recurrence and metastasis rates.

## References

[1] Thway K (2009). Pathology of soft tissue sarcomas. Clin Oncol (R Coll Radiol).

[2] Evans HL (2011). Low-grade fibromyxoid sarcoma: a clinicopathologic study of 33 cases with long-term follow-up. Am J Surg Pathol.

[3] Folpe AL, Lane KL, Paull G, Weiss SW (2000). Low-grade fibromyxoid sarcoma and hyalinizing spindle cell tumor with giant rosettes: a clinicopathologic study of 73 cases supporting their identity and assessing the impact of high-grade areas. Am J Surg Pathol.

[4] Evans HL (1993). Low-grade fibromyxoid sarcoma. A report of 12 cases. Am J Surg Pathol.

[5] Goodlad JR, Mentzel T, Fletcher CD (1995). Low grade fibromyxoid sarcoma: clinicopathological analysis of eleven new cases in support of a distinct entity. Histopathology.

[6] van den Bossche MR, Van Mieghem H (2000). Low-grade fibromyxoid sarcoma. Oncology.

[7] Oda Y, Takahira T, Kawaguchi K (2004). Low-grade fibromyxoid sarcoma versus low-grade myxofibrosarcoma in the extremities and trunk. A comparison of clinicopathological and immunohistochemical features. Histopathology.

[8] Guillou L, Benhattar J, Gengler C (2007). Translocation-positive low-grade fibromyxoid sarcoma: clinicopathologic and molecular analysis of a series expanding the morphologic spectrum and suggesting potential relationship to sclerosing epithelioid fibrosarcoma: a study from the French Sarcoma Group. Am J Surg Pathol.

[9] Meng GZ, Zhang HY, Bu H, Geng JG (2009). Low-grade fibromyxoid sarcoma versus fibromatosis: a comparative study of clinicopathological and immunohistochemical features. Diagn Cytopathol.

[10] Singh K, Singh S, Pal N, Sampley SK, Chhabra K (2012). Low-grade fibromyxoid sarcoma of anterior abdominal wall. Indian J Surg.

[11] Hashimoto M, Koide K, Arita M, Kawaguchi K, Mikuriya Y, Iwata J, Iwamoto T (2016). A low-grade fibromyxoid sarcoma of the internal abdominal oblique muscle. Case Rep Surg.

[12] Sakaguchi T, Hamada Y, Nakamura Y, Shirai T, Hamada H, Kon M (2016). Low-grade fibromyxoid sarcoma of the abdominal wall in an 8-year-old boy. J Pediatr Surg Case Rep.

[13] Ud Din N, Ahmad Z, Zreik R, Horvai A, Folpe AL, Fritchie K (2018). Abdominopelvic and retroperitoneal low-grade fibromyxoid sarcoma: a clinicopathologic study of 13 cases. Am J Clin Pathol.

[14] Ronen S, Ko JS, Rubin BP (2023). Superficial low-grade fibromyxoid sarcoma. J Cutan Pathol.

